# How an Online Intervention to Prevent Excessive Gestational Weight Gain Is Used and by Whom: A Randomized Controlled Process Evaluation

**DOI:** 10.2196/jmir.3483

**Published:** 2014-08-20

**Authors:** Margaret Mochon Demment, Meredith Leigh Graham, Christine Marie Olson

**Affiliations:** ^1^Community NutritionDivision of Nutritional SciencesCornell UniversityIthaca, NYUnited States

**Keywords:** online intervention, obesity prevention, latent class analysis, socioeconomic differences, demographic subgroups, online engagement, process evaluation

## Abstract

**Background:**

Online interventions have emerged as a popular strategy to promote healthy behaviors. Currently, there is little agreement about how to capture online intervention engagement. It is also uncertain who engages with weight-related online interventions and how engagement differs by demographic and weight characteristics.

**Objective:**

The objectives of this study were to (1) characterize how pregnant women engaged with features of an online intervention to prevent excessive gestational weight gain, (2) identify demographic and weight status subgroups of women within the sample, and (3) examine differences in use of intervention features across the demographic and weight status subgroups.

**Methods:**

A sample of racially and socioeconomically diverse pregnant women from a northeastern US city was assigned to the intervention group in a randomized controlled trial to prevent excessive gestational weight gain (n=1014). The intervention website included these features: weight-gain tracker, health-related articles, blogs, physical activity and diet goal-setting tools, and local resources. Engagement variables were created to capture the amount, consistency, and patterns of feature use across pregnancy using latent class analysis. Demographic/weight status subgroups were also created using latent class analysis. Differences in engagement across the demographic/weight status subgroups were examined using chi-square analysis.

**Results:**

Six engagement patterns emerged: “super-users” (13.02%, 132/1014), “medium-users” (14.00%, 142/1014), “consistent weight-tracker users” (14.99%, 152/1014); “almost consistent weight-tracker users” (21.99%, 223/1014), “inconsistent weight-tracker users” (15.98%, 162/1014), and “non-users” (20.02%, 203/1014). Four demographic/weight status subgroups emerged: three minority and one white. There were different engagement patterns by demographic/weight status subgroups. Super-users were more likely to be in the white subgroup, while non-users were more likely to be in the minority subgroups. However, around a third of women in minority subgroups were consistently or almost consistently engaging with the weight-tracker (black, young women, 32.2%, 67/208; black, heavier women, 37.9%, 50/132; Hispanic women, 27.4%, 32/117).

**Conclusions:**

While white and higher income women had higher engagement in general, depending on the measure, there was still considerable engagement by the minority and low-income women.

**Trial Registration:**

Clinicaltrials.gov: NCT01331564; http://clinicaltrials.gov/ct2/show/NCT01331564 (Archived by WebCite at http://www.webcitation.org/6Rw4yKxI5).

## Introduction

Online interventions have emerged as a popular strategy in obesity prevention. In order to understand the effectiveness of these interventions, it is critical to identify how participants use online interventions and if there are differences by demographic characteristics and weight status. Process evaluation is an important step for understanding how interventions achieve their intended outcomes. A key process measure is dose of intervention received. A higher dose of intervention received or higher engagement has generally been associated with greater success in achieving weight-related intervention outcomes [1-3]. This has been particularly true in online behavior change interventions [4-6]. While online interventions provide a unique opportunity to measure engagement objectively, there is no current consensus on the definitions and measures for engagement in online interventions [7]. Previous studies have used the following measures of use: number of website visits or log-ins; time spent on a site; and number of features used [7-9]. No studies to date have examined feature use based on expected use, consistency of use across the intervention time period, or how usage clusters across different intervention features.

In this study, online engagement with a website to prevent excessive gestational weight gain (GWG) is examined. Avoiding excessive GWG, defined as gaining more than the Institute of Medicine’s GWG recommendations, has become a priority in obesity prevention [10]. Excessive GWG is a risk factor for postpartum weight retention that contributes to long-term weight gain [11]. Low-income and minority women are more likely to be overweight and obese and are more likely to gain more weight than recommended during pregnancy [12]. Given this increased risk for excessive GWG, it would be valuable to know how low-income and minority women engage with an online intervention. Differences in engagement for GWG interventions by demographic characteristics have not been examined. However, rates of participation in diabetes self-management trials were highly variable in diverse samples inclusive of lower income individuals and minorities [13]. In addition, in a 2-year weight loss trial, obese non-white participants were significantly less likely to self-weigh weekly compared to white participants [13,14].

In most studies, demographic and weight status variables are examined independently and statistical methods isolate the independent effect of each factor. This has several methodological challenges as articulated by Lanza et al [15] and summarized here: (1) it can lead to Type 1 error (eg, the need for multiple comparisons to be run on each characteristic increasing the likelihood of finding a significant result), (2) the statistical power to detect an effect varies by characteristics (depending on the number of individuals within the categories for the characteristic), and (3) higher order interactions are often impossible to evaluate due to sample size constraints (eg, comparing older white females to younger white females) [16]. For this study, we use an alternative method that has emerged in prevention medicine [16-18]. Rather than focusing on isolated risk factors or characteristics, individuals with all their demographic characteristics and weight status are first categorized into multidimensional subgroups through latent class analysis and then the subgroup variable is used in subsequent analyses, rather than independent variables for each demographic characteristic and weight status.

This study addresses two gaps in the current understanding of weight-related online interventions. First, this study provides new measures of engagement that consider expected use, consistency of use across time, and patterns of use for different features. Second, this study examines how multiple measures of engagement differ across demographic/weight status subgroups. The aims of this study were to (1) characterize how pregnant women engaged with online intervention features, (2) identify demographic and weight status subgroups of women within the sample, and (3) examine differences in use of intervention features across the demographic and weight status subgroups.

## Methods

### Intervention

Fishbein and Yzer’s Integrative Model of Behavioral Prediction [19] was the guiding theoretical framework for the online intervention to prevent excessive gestational weight gain. Fishbein and Yzer’s framework was combined with Fogg’s Behavior Model for Persuasive Design [20] to link behaviors and their predictors to intervention features. The online intervention included blogs, local resources, articles, frequently asked questions (FAQs), and events. In addition, intervention participants also had access to the weight gain tracker and diet and physical activity goal-setting tools. Intervention participants were emailed weekly from randomization to delivery with new content and reminders to use the weight gain tracker, diet, and physical activity goal-setting tools. Intervention features are described in more detail in Graham et al 2014 [21] and images are available in Multimedia Appendix 1.

### Participants

Data from the intervention group of a randomized controlled trial of excessive GWG and postpartum weight retention prevention with women who were 18-35 years of age, normal to obese class I body mass index (BMI), socially and racially diverse, and relatively healthy (N=1689), conducted in the northeastern United States, were used in this study. That randomized trial, conducted from 2011-2014, is described in detail elsewhere [22,23]. To meet eligibility criteria, participants had to (1) consent at or before 20 weeks gestation, (2) be available for a 24-month intervention, (3) plan to carry their pregnancy to term and keep their baby, (4) read and understand English, and (5) have an email address. Exclusion criteria included: BMI<18.5 kg/m^2^(underweight) or >35.0 kg/m^2^(class 2 obese), multiple gestation (eg, twins), having had eating disorders or gastric bypass surgery in the past, having had three or more consecutive miscarriages, and the presence of pre-pregnancy medical conditions that could influence weight loss or gain. All study participants were sent an email describing the tools on the website, and email, a postcard, and telephone reminders were used as prompts to encourage participants to visit the website the first time. A US$5 incentive was also given for the first website visit. There were 1126 eligible women who were assigned to the intervention group for pregnancy. The sample for this analysis included women who participated in the study through pregnancy (ie, did not withdraw, miscarry, or have a pre-term birth at less than 28 weeks gestation) (n=1014). For this analytic sample, participants were exposed to the intervention for a minimum of 2 months and a maximum of 9 months depending on both week gestation at enrollment and week gestation at delivery. The women excluded from analysis did not significantly differ from those included (Table 1).

**Table 1:** Sample characteristics.

Characteristic		Total sample, n=1689 n (%)	Intervention sample, n=1126 n (%)	Analysis sample, n=1014 n (%)	*P* value^a^
**Race**					.28
	White	1054 (62.40)	693 (61.55)	630 (62.13)	
	Black	395 (23.39)	273 (24.25)	239 (23.57)	
	Other	240 (14.21)	160 (14.21)	145 (14.30)	
**Hispanic**					.44
	Yes	212 (12.55)	145 (12.88)	128 (12.62)	
	No	1477 (87.45)	981 (87.12)	886 (87.38)	
**Low-income**					.57
	Yes	734 (43.46)	494 (43.87)	442 (42.59)	
	No	955 (56.54)	632 (56.13)	572 (56.41)	
**Body mass index category**					.74
	Normal	872 (51.63)	575 (51.07)	520 (51.28)	
	Overweight	508 (30.08)	346 (30.73)	308 (30.37)	
	Obese	309 (18.29)	205 (18.21)	186 (18.34)	
**Age category, years**					.90
	18 - <25	506 (29.96)	341 (30.28)	305 (30.08)	
	25 - <30	546 (32.33)	363 (32.24)	328 (32.35)	
	≥30 years	637 (37.71)	422 (37.48)	381 (37.57)	

^a^Chi-square analysis *P* value comparing analysis sample and those not included (n=112) from the intervention sample.

### Data Collection

Five sources of data were used in this analysis: screening for eligibility, postpartum height and weight visit, medical chart audit, website activity, and survey. At baseline screening, which took place at less than 20 weeks gestation, the following self-reported variables were collected: race, ethnicity, date of birth, height, current weight, early pregnancy (<13 weeks) or pre-pregnancy weight, and a measure of low-income using a participant’s insurance type to determine if a participant qualified for Women, Infants and Children Program (WIC)/ Medicaid/ Prenatal Care Assistance Program (PCAP). The self-reported ethnicity question asked, “Are you of Hispanic or Latino origin?” with the response categories of “yes” and “no”. The self-reported race item asked, “Which race best describes you?” with the response categories of “American Indian and Alaska Native”, “Asian”, “Black or African American”, “White”, “Native Hawaiian and other Pacific Islander”, and “Other race, please specify”.

To categorize women’s weight status, measured weight and height were used to calculate BMI for the vast majority of the sample. Height was collected from three data sources: (1) measured height from postpartum weight collection visits (1307/1689, 77.38% of sample), (2) measured height from the medical chart (358/1689, 21.20% of sample), or (3) self-reported height at screening (24/1689, 1.42% of sample). Pre-pregnancy or early pregnancy weight was collected from three data sources: (1) measured early pregnancy weight from the medical chart (1599/1689, 94.67%), (2) self-reported or measured pre-pregnancy weight from the medical chart (67/1689, 3.97%), or (3) self-reported pre-pregnancy or early pregnancy weight at screening (23/1689, 1.36% of sample).

Medical chart audit data were used to verify and correct the date of birth of the participant (33/1689 individuals with changed date of births, 1.95% of total study sample). Date of birth and date of consent were used to calculate age of the subject at time of entry into the study.

Each participant’s online activities were continuously collected throughout the study automatically by the website. Each website activity was time stamped and only activities from consent date to delivery date were included in this analysis. All activities associated with intervention features, rather than data collection activities such as surveys, were considered intervention use in this analysis.

All randomized participants were asked to complete a baseline questionnaire. The questionnaire was available online and via telephone from consent date and until the participant was greater than 28 weeks into pregnancy. A survey item that asked about home Internet use was included in this research.

### Conceptualizing Measures of Engagement

Use of the following six intervention features were used to characterize engagement: health-related information (articles and FAQs), blogs, local resources, diet goal-setting tools, physical activity goal-setting tools, and a weight-gain tracker. Features were categorized based on expected use. Consistent use was expected for log-ins and entry of weights into the weight gain tracker. We expected women to track their weight in 30-day intervals but, to allow for difference in timing of doctor’s visits, we created 45-day intervals from time of enrollment to delivery. If a woman entered a weight during each of the 45-day intervals that she completed, she was categorized as a “consistent tracker”. If during at least of half of the intervals a woman entered a weight, she was categorized as an “almost consistent tracker”. If a woman had entered at least one weight but not during more than half of her intervals, she was categorized as an “inconsistent tracker”. Finally, if she never entered a weight during pregnancy, she was categorized as a “non-weight tracker”. The same procedure was used to categorize use for log-ins.

For all other features, consistent use was not expected. Use was expected on an “as needed” basis. Therefore, quantity of use defined engagement for the following features: health-related information, blogs, resources, diet goal-setting tools, and physical activity goal-setting tools. A woman’s engagement was categorized into three levels for each of these features: “high” (≥median among users), “low” (<median among users), or “never” (0).

### Demographic Subgroups

Since sociodemographic characteristics are correlated and most sociodemographic characteristics are measured categorically, several recent studies have employed latent class or subgroup analysis to group women with similar characteristics together [15,17,18,24]. Demographic/BMI subgroups were created in the analysis sample (n=1014) based on the following variables: race (white, black, or other), ethnicity (Hispanic or non-Hispanic), low-income status (<185% poverty line or ≥185% poverty line), BMI category (normal (BMI 18-<25), overweight (BMI 25-<30), or obese class 1 (BMI 30-<35), and age category (18-<25 years, 25-<30 years, or 30-35 years).

### Home Internet Use

The data for home Internet usage came from the baseline questionnaire survey item: “How often do you access the Internet from your home?” and had the following response categories: never; less than once a week; a few times a week; most days of the week; every day (859/1014, 84.71% of analytic sample). For the purposes of this analysis, we used the following categorizations: “never/occasionally” (never to a few times a week), “most days of the week”, and “every day”.

### Analysis

#### Engagement Patterns

Latent class analysis (LCA) was used to identify patterns of feature use as a measure of overall intervention engagement [25]. Often the latent class variable is used to organize multiple dimensions of behavior, such that individuals in each latent class share common behavior patterns. In our case, this analysis was used to group individuals based on their similar patterns feature use of the intervention website.

LCA models are fit in a series of steps starting with a one-class model; the number of classes is subsequently increased until there is no further improvement in the model. Model selection in LCA involved both absolute fit of a particular model and relative fit of two or more competing models. A common measure of absolute model fit in categorical models is the G^2^ likelihood-ratio chi-square statistic, which in our case tests the null hypothesis that the specified LCA model fits the data [26]. Relative fit of models with different numbers of latent classes (eg, 4 vs 5 classes) was analyzed using a series of standard fit indices, including the Bayesian information criterion (BIC [27]) and Akaike information criteria (AIC [28]), with a lower value suggest a more optimal balance between model fit and parsimony. All analyses were conducted using a SAS procedure, PROC LCA [16].

#### Demographic/Body Mass Index Subgroups

LCA was used to identify demographic/BMI subgroups. Given the strong correlation between demographic and BMI characteristics in this sample, we decided to take a person-centered approach to categorizing women. To do this, we used LCA to identify subgroups within the population based on race, ethnicity, income, BMI, and age. The same LCA model selection criteria were used as with the engagement patterns outlined above.

#### Association Between Demographic/Body Mass Index Subgroups and Engagement

Chi-square analysis was used to first examine the relationship between individual feature use and demographic subgroup. Next, chi-square analysis was used to examine the relationship between demographic subgroups and patterns of engagement.

The data analysis for this paper was generated using SAS software, version 9.3.

## Results

### Characterize How Pregnant Women Engaged With Online Intervention Features

The first objective of this study was to capture multiple measures of *how* women used the intervention website. Most women logged into the website during pregnancy (87.97%, 892/1014) and engaged with the intervention features. As described earlier, consistency of use or quantity of use was used to characterize dose for each intervention feature. Of the intervention features, the weight tracker was most commonly used with 25.05% (254/1014) of women who consistently used, 28.99% (294/1014) almost consistently used, 19.03% (193/1014) inconsistently used, and 26.04% (264/1014) never used (Table 2). Health-related information and blogs were engaged by over half of the sample, while the diet and physical activity goal-setting tools were utilized by only a third of the sample.

When all intervention features were considered together, six patterns of engagement emerged from the latent class analysis, as shown in the column headings in Table 3. The first class was characterized by high and consistent usage of all features and is labeled “super-users” (13.02%, 132/1014). “Medium-users” (14.00%, 142/1014) were characterized by almost consistent weight-tracker use and high use of both health-related information and blogs. The next three classes were characterized in the latent class analysis solely based on weight-tracker use: “consistent weight-tracker users” (14.99%, 152/1014); “almost consistent weight-tracker users” (21.99%, 223/1014), and “inconsistent weight-tracker users” (15.98%, 162/1014). The final class, “non-users” (20.02%, 203/1014) were categorized by never engaging with the intervention features.

**Table 2:** Proportion of total sample (n=1014) that used website features.

Feature use			Analysis sample n (%)
**Feature categorized by consistency**			
	**Log-in**		
		Consistent	332 (32.74)
		Almost consistent	342 (33.73)
		Inconsistent	214 (21.11)
		Never used	126 (12.42)
	**Weight-tracker**		
		Consistent	252 (24.85)
		Almost consistent	298 (29.39)
		Inconsistent	196 (19.33)
		Never used	268 (26.43)
**Feature categorized by quantity**			
	**Health-related info**		
		High	270 (26.63)
		Low	229 (22.58)
		None	515 (50.79)
	**Blogs**		
		High	277 (27.32)
		Low	272 (26.82)
		None	465 (45.86)
	**Resources**		
		High	207 (20.41)
		Low	175 (17.26)
		None	632 (62.33)
	**Physical activity goal setting**		
		High	176 (17.36)
		Low	139 (13.71)
		None	699 (68.93)
	**Diet goal setting**		
		High	182 (17.95)
		Low	142 (13.81)
		None	690 (68.04)

**Table 3:** Patterns of intervention feature use identified from latent class analysis probabilities.

Feature use		Super-users	Medium-users	Weight-consistent	Weight-almost consistent	Weight- inconsistent	Non-users
		(13.02%, 132/1014)	(14.00%, 142/1014)	(14.99%, 152/1014)	(21.99%, 223/1014)	(15.98%, 162/1014)	(20.02%, 203/1014)
**Log-in**							
	Consistent	0.93	0.12	0.97	0.14	0.04	0.00
	Almost consistent	0.07	0.86	0.03	0.84	0.08	0.02
	Inconsistent	0.00	0.02	0.00	0.01	0.89	0.34
	Never	0.00	0.00	0.00	0.00	0.00	0.64
**Weight-tracker**							
	Consistent	0.88	0.00	0.83	0.02	0.00	0.00
	Almost consistent	0.11	0.85	0.13	0.63	0.00	0.00
	Inconsistent	0.01	0.13	0.01	0.28	0.71	0.00
	Never	0.00	0.02	0.03	0.08	0.29	1.00
**Physical activity goal setting**							
	High	0.53	0.45	0.18	0.02	0.04	0.00
	Low	0.17	0.21	0.14	0.14	0.20	0.00
	None	0.30	0.33	0.67	0.84	0.76	1.00
**Diet goal setting**							
	High	0.50	0.42	0.17	0.05	0.10	0.00
	Low	0.16	0.20	0.18	0.15	0.20	0.00
	None	0.34	0.38	0.65	0.80	0.70	1.00
**Health-related info**							
	High	0.84	0.65	0.23	0.08	0.06	0.00
	Low	0.16	0.27	0.42	0.31	0.20	0.01
	None	0.00	0.09	0.35	0.61	0.73	0.99
**Blogs**							
	High	0.91	0.59	0.24	0.09	0.08	0.00
	Low	0.07	0.31	0.44	0.43	0.33	0.01
	None	0.01	0.09	0.32	0.48	0.59	1.00
**Local resources**							
	High	0.89	0.47	0.06	0.01	0.05	0.00
	Low	0.11	0.27	0.33	0.20	0.16	0.00
	None	0.00	0.25	0.61	0.79	0.80	1.00

### Identify Demographic/Body Mass Index Subgroups of Pregnant Women

The second objective was to create a holistic measure for demographic/BMI characteristics through latent class analysis. From this analysis, four demographic/BMI subgroups emerged, characterized primarily by race, ethnicity, and income (Table 4). The largest subgroup comprised mainly white, non-Hispanic, not low-income, normal weight, and 30 years or older women (54.93% of the sample, 557/1014), which for brevity has been labeled the “white” subgroup. This subgroup was the only subgroup with a high probability of being higher income and older. There were two subgroups that had a high probability of being black. The first comprised black women who were predominantly non-Hispanic, low-income, normal weight, and 18 to 25 years old (20.51%, 208/1014) and is termed “black, young”. The second subgroup comprised black women and was also predominantly non-Hispanic and low-income, but differed by BMI. In this subgroup, women were more likely to be overweight or obese BMI (13.02%, 132/1014). This subgroup was labeled “black, heavier” to denote the distinction between the two subgroups that had a high likelihood of being black. The final group comprised predominantly Hispanic women who were also likely to be low-income, normal, or overweight BMI, and 18 to 25 years old (11.54%, 117/1014). It was the only subgroup that emerged with a greater than 50% probability of Hispanic ethnicity and as such it is labeled “Hispanic”.

**Table 4:** Demographic/body mass index (BMI) subgroups identified from latent class analysis probabilities.

		Demographic/BMI Subgroup			
		Black, young (20.51%, 208/1014)	Black, heavier (13.02%, 132/1014)	Hispanic (11.54%, 117/1014)	White (54.93%, 557/1014)
**Race**					
	White	0.29	0.42	0.02	0.94
	Black	0.71	0.56	0.01	0.00
	Other	0.00	0.01	0.96	0.06
**Hispanic**					
	Yes	0.06	0.04	0.84	0.02
	No	0.94	0.96	0.16	0.98
**Low-income**					
	Yes	0.89	0.67	0.78	0.11
	No	0.11	0.33	0.22	0.89
**Body mass index category**					
	Normal	0.67	0.12	0.41	0.60
	Overweight	0.20	0.45	0.41	0.27
	Obese	0.13	0.43	0.18	0.12
**Age category**					
	18 - <25	0.82	0.23	0.55	0.07
	25 - <30	0.14	0.49	0.31	0.34
	≥30	0.03	0.28	0.15	0.59

### Examine Differences in Use of Intervention Features Across Demographic/Body Mass Index Subgroups

In the final objective, we examined *who,* based on the demographic/BMI subgroups, engaged with the online intervention. Use of all intervention features was significantly different across demographic/BMI subgroup (Table 5).

The weight-tracker was used by more than half of each of the demographic subgroups. The predominantly white subgroup had the smallest proportion of women who never used it (12.6%, 70/557) and the highest proportion of women who used it consistently (35.7%, 199/557). The minority subgroups also used the weight-tracker but to a lesser degree.

**Figure 1:**
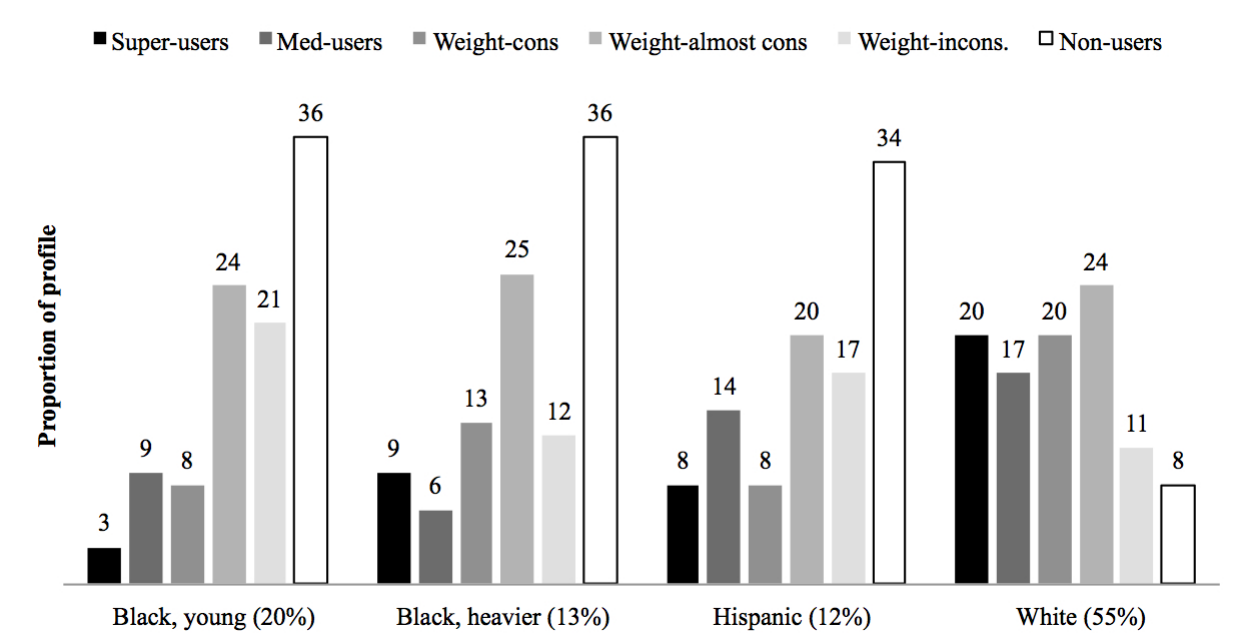
Associations between patterns of online engagement and demographic/body mass index (BMI) subgroups (n=1014).

When comparing use of the intervention features together with the LCA patterns, there were pronounced differences across demographic/BMI subgroups (Figure 1). Super-users were more likely to be in the white subgroup compared to other subgroups (20% vs 3%, 9%, and 8%), while non-users were more likely to be in the minority subgroups compared to other subgroups (36%, 36%, and 34% vs 8%). However, around a third of individuals in the minority subgroups were consistently or almost consistently engaging with the weight-tracker (black, young women, 32.2%, 67/208; black, heavier women, 37.9%, 50/132; Hispanic women, 27.4%, 32/117).

**Figure 2:**
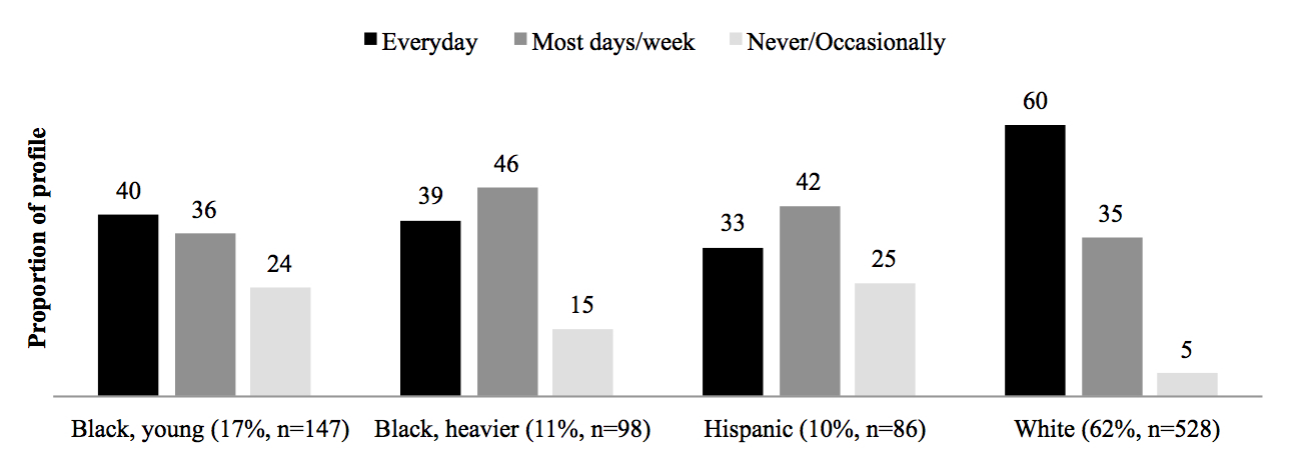
Frequency of home Internet use by demographic/body mass index subgroup (84.71%, 859/1014 women in the analysis sample completed the survey question regarding home Internet use).

While home Internet use behaviors varied across demographic/BMI subgroups (Figure 2), at least 75% of each subgroup used the Internet every day or most days of the week. However, only 26 out of 528 (4.9%) of women in the white subgroup rarely or never used the Internet, while 35 out of 147 (23.8%) of women in the black, young subgroup; 15 out of 98 (15%) of women in the black, heavier subgroup; and 26 out of 86 (25%) of women in the Hispanic subgroup rarely or never used the Internet at home.

**Table 5:** Intervention feature use by demographic/body mass index (BMI) subgroups.

Feature use		Demographic/BMI Subgroup				*P* value^a^
		Black, young, n=208	Black, heavier, n=132	Hispanic, n=117	White, n=557	
		n (%)	n (%)	n (%)	n (%)	
**Log-in**						<.01
	Consistent	33 (15.9)	28 (21.2)	28 (23.9)	243 (43.6)	
	Almost consistent	61 (29.3)	39 (29.6)	33 (28.2)	209 (37.5)	
	Inconsistent	64 (30.8)	35 (26.5)	31 (26.5)	84 (15.1)	
	Never used	50 (24.0)	30 (22.7)	25 (21.4)	21 (3.8)	
**Weight-tracker**						<.01
	Consistent	17 (8.2)	16 (12.1)	20 (17.1)	199 (35.7)	
	Almost consistent.	40 (19.2)	30 (22.7)	27 (23.1)	201 (36.1)	
	Inconsistent	58 (27.9)	28 (21.2)	23 (19.7)	87 (15.6)	
	Never used	93 (44.7)	58 (43.9)	47 (40.2)	70 (12.6)	
**Health-related info**						<.01
	High	22 (10.6)	19 (14.4)	17 (14.5)	212 (38.1)	
	Low	30 (14.4)	25 (18.9)	25 (21.4)	149 (26.8)	
	None	156 (75.0)	88 (66.7)	75 (64.1)	196 (35.2)	
**Blogs**						<.01
	High	31 (14.9)	25 (18.9)	22 (18.8)	199 (35.7)	
	Low	44 (21.2)	35 (26.5)	23 (19.7)	170 (30.5)	
	None	133 (63.9)	72 (54.6)	72 (61.5)	188 (33.8)	
**Resources**						<.01
	High	13 (6.3)	22 (16.7)	12 (10.3)	160 (28.7)	
	Low	26 (12.5)	22 (16.7)	18 (15.4)	109 (19.6)	
	None	169 (81.3)	88 (66.7)	87 (74.4)	288 (51.7)	
**Physical activity goal setting**						<.01
	High	21 (10.1)	20 (15.2)	11 (9.4)	124 (22.3)	
	Low	21 (10.1)	19 (14.4)	15 (12.8)	84 (15.1)	
	None	166 (79.8)	93 (70.5)	91 (77.8)	349 (62.7)	
**Diet goal setting**						.009
	High	26 (12.5)	21 (15.9)	17 (14.5)	118 (21.2)	
	Low	23 (11.1)	13 (9.9)	19 (16.2)	87 (15.6)	
	None	159 (76.4)	98 (74.2)	81 (69.2)	352 (63.2)	

^a^ Chi-square analysis, *P* value comparing analysis sample and those not included (n=112) from the intervention sample.

## Discussion

### Principal Findings

#### Summary

This study examined a variety of engagement measures, based on both how participants used intervention features on the website and expected use of some features, and created data-driven measures of engagement. In addition, subgroups were identified, acknowledging that demographic and weight status characteristics are often associated with each other, allowing for a more person-centered approach. The contributions of this study by aim are outlined in the following sections.

#### Characterizing and Measuring Engagement

Two methodological contributions were made by the analysis of the online engagement in this study to prevent excessive weight gain in pregnancy. First, consistency of use over time, particularly for weight gain self-monitoring, was expected to be related to appropriate weight gain over time [29-31]. A simple count of 10 weights tracked without taking time into account could mean a woman entered all 10 weights in her first 30 days of the study and discontinued weight monitoring after that. Use of the weight gain tracker was expected to align with each prenatal visit, which typically occurs once a month during the first and second trimesters and bi-weekly in the third trimester. One weight entry per 45-day interval was used to define consistent use across time to allow for variability when participants’ doctor’s appointments could fall. Characterizing engagement by consistency of use is a unique contribution to capturing online engagement.

Second, by examining the patterns of feature use with latent class analysis, we made a novel contribution to how online interventions could measure engagement. Conceptualizing engagement as patterns rather than independent feature use was conceptually relevant in two ways. First, the website was designed to integrate features by related content. For example, if a woman read a blog about breastfeeding, links to articles or FAQs about breastfeeding appeared next to the blog. Second, the website was designed to offer at least a few features that each participant would use and it was expected that some women would engage with everything (super-users) while others might find only one or two features helpful [21]. The use of latent class analysis allowed us to examine the patterns of usage that emerged from actual use. The results of this study are similar to other studies in that a “super-user” group emerged from our latent class analysis [32-34]. These are individuals who engage with all features in high amounts. The findings of this study move beyond just identifying super-users to identify clustering of feature use at varying intensities.

Interestingly, consistency of weight tracking was a defining feature across most of the patterns. The weight tracking tool was one of the most used features of the website and it was used by a variety of demographic/BMI subgroups. Around a third of the minority subgroups were consistently or almost consistently engaging with the weight-tracker (black, young women, 32%; black, heavier women, 38%; Hispanic women, 28%). The implication for future online interventions is that in order to reach a diverse sample, online interventions need to offer a variety of features and need to acknowledge that engagement in the intervention will vary.

An additional consideration for this analysis is that count data, which could be considered continuous, is challenging to use with parametric methods like factor analysis due to the non-normal distribution of the data [35]. This skewness of engagement data is common to online interventions and is typically handled in other studies by counting use of a feature as ever use or never use for both the use of ever and never use [21,32,36-38]. By categorizing the usage of each feature into no use, low use, and high use, we captured some of the spectrum of use for “as needed” features (eg, blogs, articles, resources) and avoided the challenges of dealing with non-normal data with continuous methods.

#### Subgroups

Utilizing latent class analysis for creating participant subgroups [17] is a relatively novel approach for examining socioeconomic characteristics of individuals. Utilizing a subgroup analysis methodology like latent class analysis for demographic and weight characteristics, which are known to be correlated, allows for a more holistic characterization of individuals. This analysis sought to understand the website use behaviors of the woman considered more comprehensively, taking into account race, ethnicity, income, age, and BMI together.

The findings from this study suggest that minority and low-income women were less engaged with the website compared to white, higher-income women. However, we also found that a significant proportion of minority and low-income women were also engaging with the website, but not as much or as consistently.

#### Digital Divide

The digital divide, the inequity between groups in access, use, and knowledge of technology, is an important consideration for online interventions seeking to reach a social and racially diverse population. Even though there are no longer significant differences in both smartphone use and Internet use comparing minorities to whites, there are significant differences by income in Internet and smartphone use [39]. From 2000 to 2011, Internet use increased for blacks (35% to 71%) and for those earning less than US$30,000/year (28% to 62%) [39]. Yet Internet use among households that earn more than US$50,000/year is between 90-97%, while current rates among African Americans or those earning less than US$30,000/year is still much lower [39]. The results presented in this study are consistent with these numbers with about a third of the minority subgroups being non-users in the intervention compared to 8% in our white subgroup.

### Limitations

Several methodological choices were made that could be seen as limitations. First, for making the demographic/BMI subgroups, we chose to use only variables that were available for all women. This limited the variables for creating the subgroups to screening variables only. Other subgroup analyses have used up to 40 variables to group participants. Had more variables been available, this may have led to more complex subgroups. Second, the digital divide question for the sample comes from an online survey with a back-up telephone survey. While 85% of the sample answered that question, it is likely that these women were more likely to be Internet users. Third, since this study was conducted with pregnant women, its generalizability is limited particularly given their increased likelihood to seek information online compared to the general population [40].

Gestational weight gain outcomes were not examined as part of this analysis though these outcomes will be examined in relation to both treatment assignment and intervention engagement in future analyses. These future analyses will facilitate understanding whether use of particular intervention features accounts for any overall intervention effect on weight outcomes. Across subgroups there were women who never used the online intervention; exploring why some participants never engaged in the intervention is an area of research needed.

### Conclusions

Engagement in online interventions is still a concern for reaching the population of most disadvantaged pregnant women. However, in this study with a population-based sample recruited from healthcare practices, a large number of women were reached and even minority and low-income women engaged to varying degrees with the intervention. Acknowledging both the reach of an online intervention and differential engagement in an online intervention are critical to understanding and interpreting the results of current efficacy trials of online interventions and to the design of future online interventions.

## Multimedia Appendix 1

Brief description and screenshot image of intervention website features.

## Abbreviations

BMI: body mass index
GWG: gestational weight gain
LCA: latent class analysis
