# Supplementary material for: How an Online Intervention to Prevent Excessive Gestational Weight Gain Is Used and by Whom: A Randomized Controlled Process Evaluation
Source: J Med Internet Res. 2014 Aug 20;16(8):e194. doi: 10.2196/jmir.3483 (PMC4156016; doi:10.2196/jmir.3483)

MULTIMEDIA APPENDIX 1: Brief description and screenshot image of intervention website features

Intervention Participant Dashboard Screenshot

After a participant logs into the study website this is the page that she sees. New content is displayed in the live feed (including articles, FAQs, blogs and resources). Surveys that are part of the data collection activities are displayed in yellow. Weight monitoring and goal setting are prominently featured and all other website features can be accessed via the menu bar.

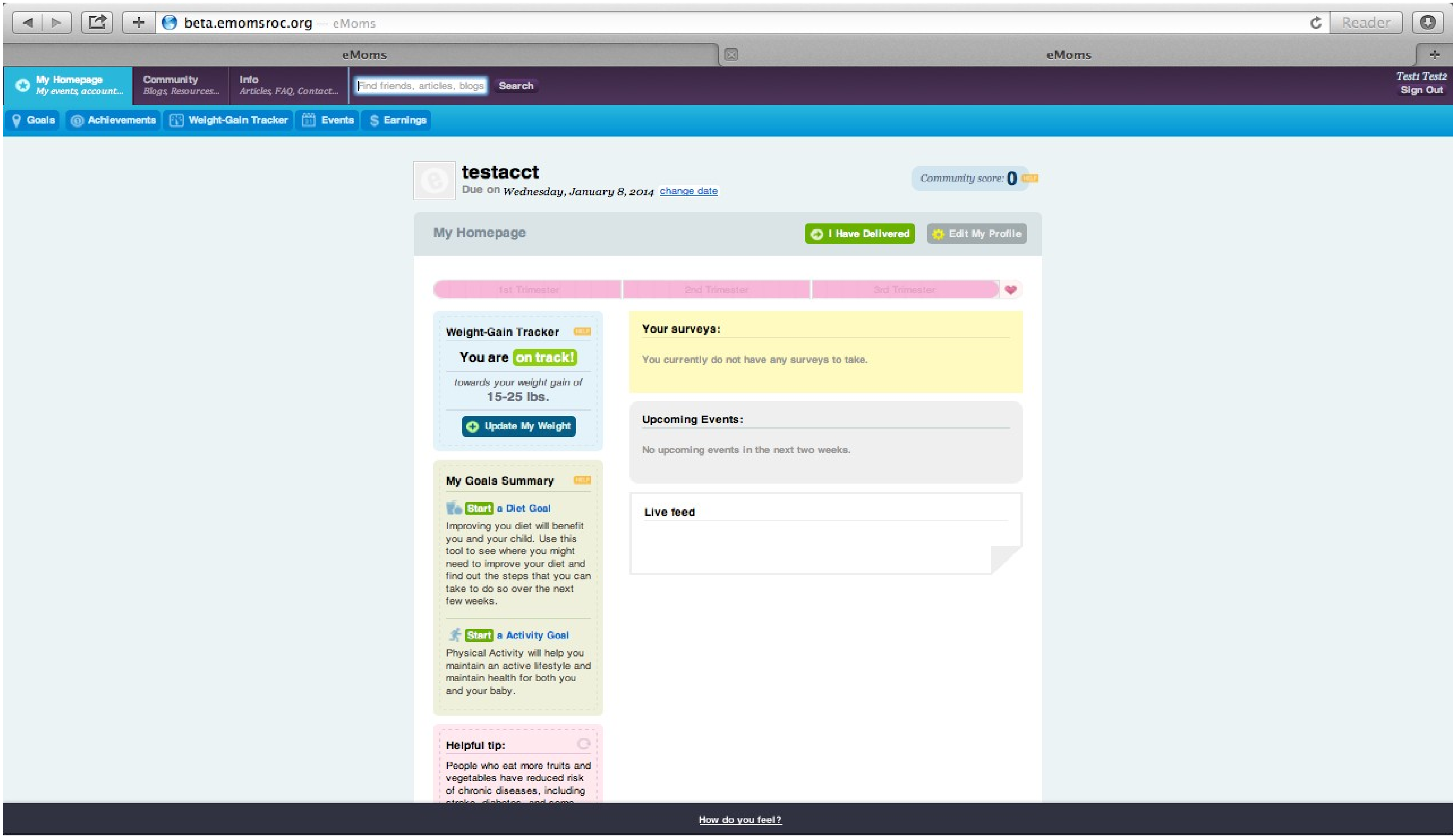

Weight Gain Tracker
This is the tracker for a woman that had a pre-pregnancy body mass index that placed her in the overweight category. The recruitment and pre-pregnancy weights were collected by study staff at enrollment and automatically plotted on the graph. The other weight was entered by the participant. Only the weights entered by participants are included in the engagement monitoring.

Weight-Gain Tracker HELP

You are on track!

towards your weight gain of

15-25 lbs.

Update My Weight

Use the Weight Gain Tracker to enter your weight throughout your pregnancy to keep track of the weight that you are gaining. **Try to stay within the shaded area!** If you do, then you will be on track to gain the right amount of weight for this pregnancy.

Adequate weight gain during pregnancy reduces the risk of having a low birth weight baby. Too much weight gain increases your risk of being overweight after birth. You should try to stay in your Weight Gain Range of **15-25 .lbs**

Your current weight gain progress:

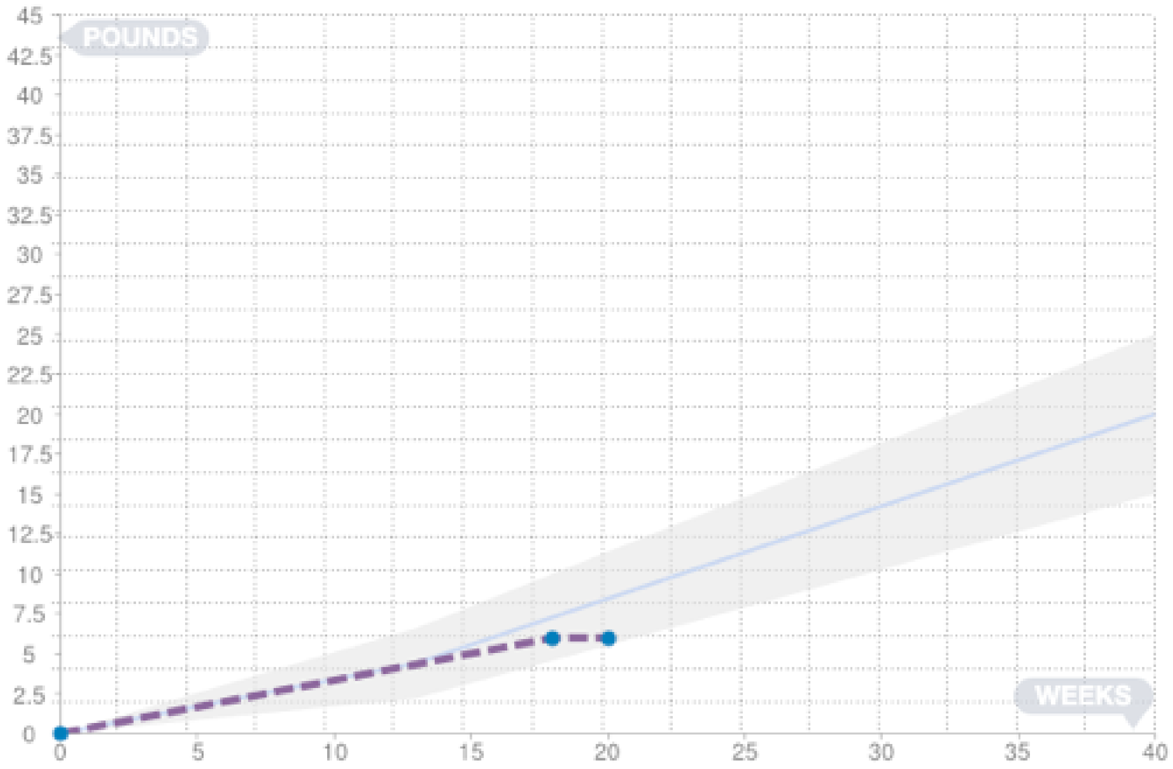

Start Weight gathered at recruitment: ***154.0 lbs***

Weight Gain History

Date	Weight	
Friday, August 23, 2013	160.0	Edit Delete
Saturday, August 10, 2013 (recruitment weight)	160.0	
Wednesday, April 3, 2013 (pre-pregnancy weight)	154.0	

Goal-Setting Tools: More Information Screen
For each feature on the website there is a more information pop-out display. They were included to provide an overview of each feature.

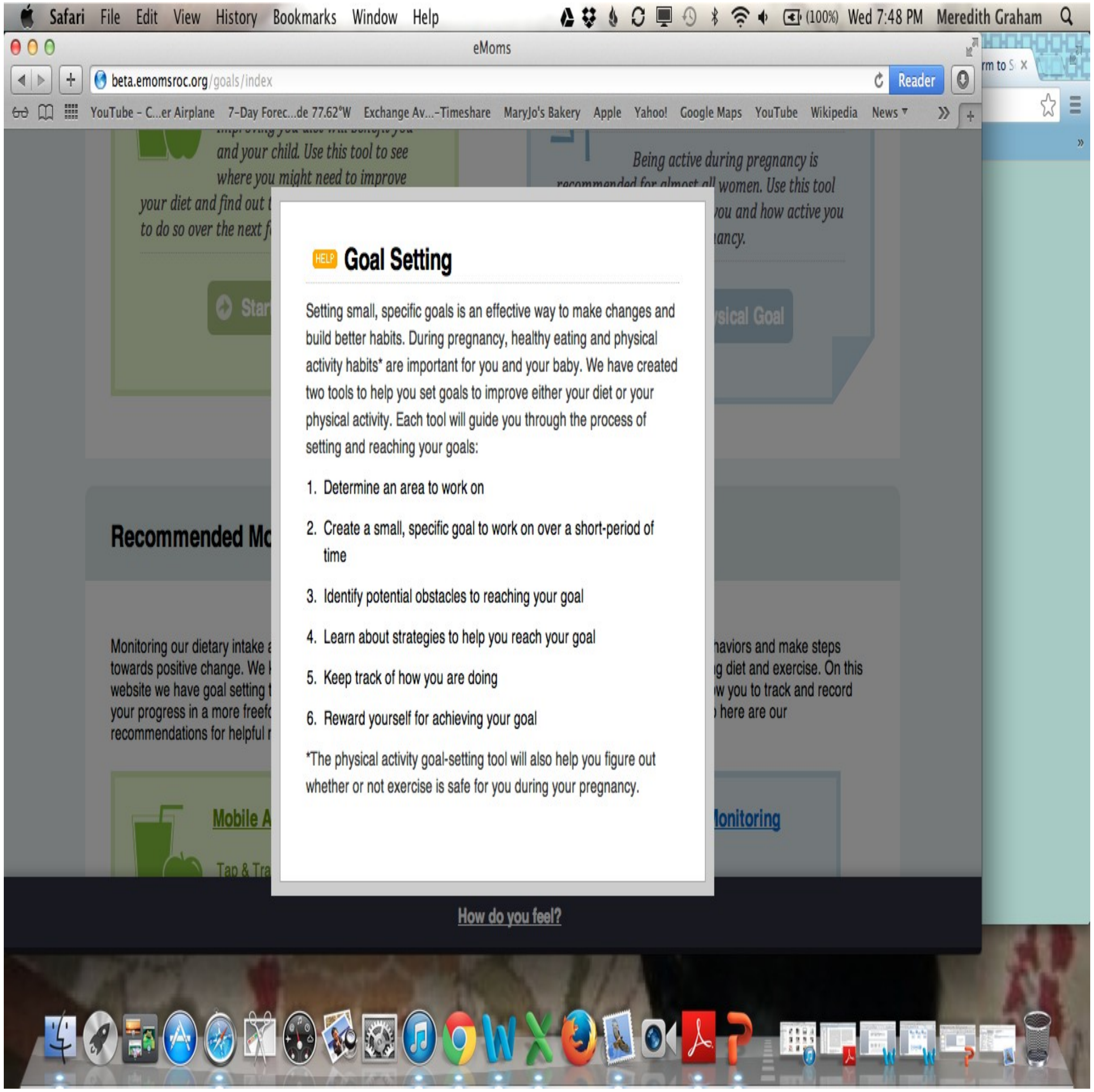

Diet Goal-Setting Self-Monitoring
After a participant has set a goal she is encouraged to monitor her goal. Information and social support through blogs that are related to the selected goal are displayed along the left side of the self-monitoring page.

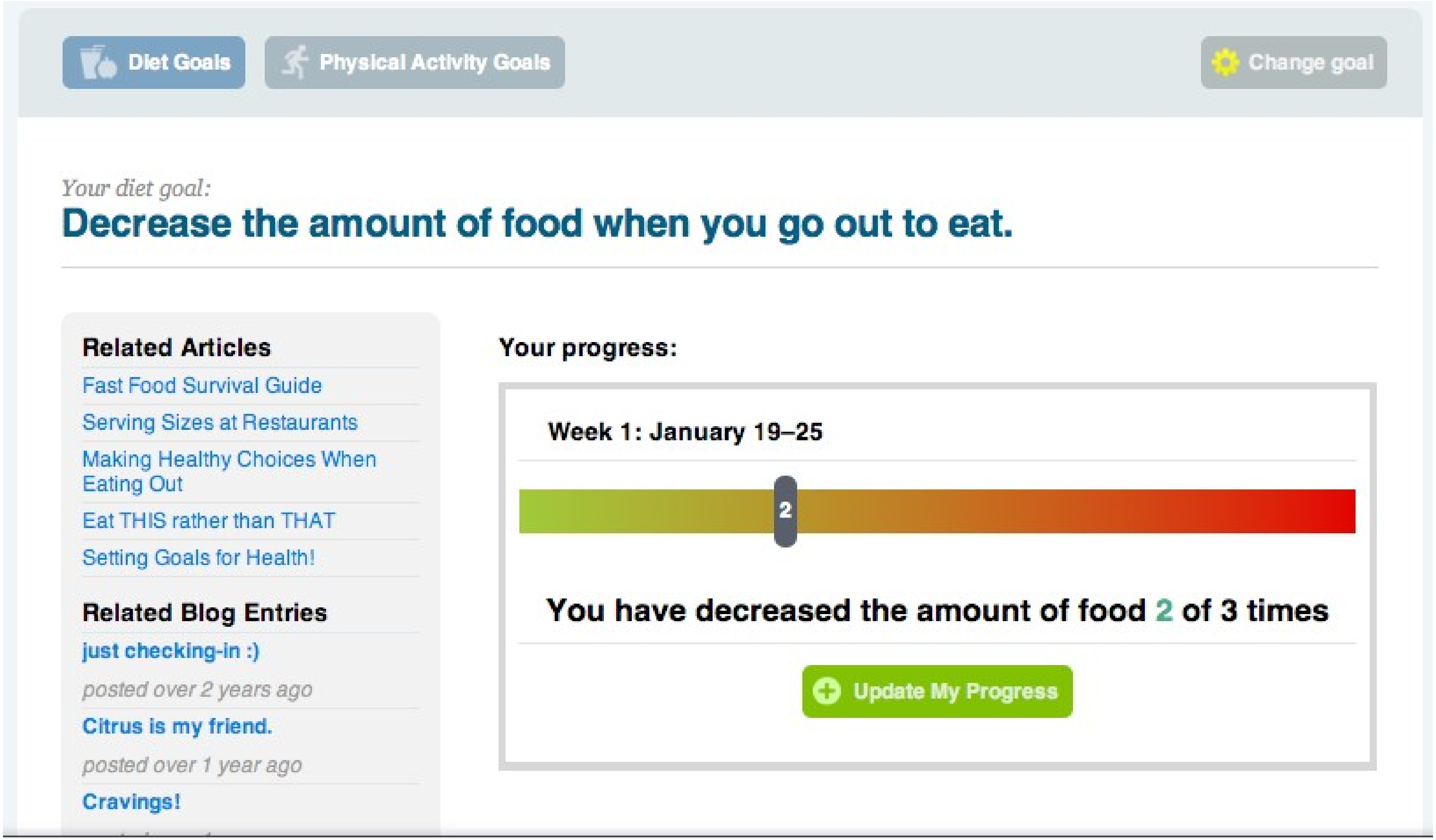

Information Section
Health related articles and frequently asked questions are included in the information section related to nutrition, physical activity, gestational weight gain, maternal health and infant health.

Info:

Popular Articles

How Much Sleep Is Enough?
Posted over 1 year ago & has 3 comments

Body Changes and Discomforts: Fatigue and Sleep Problems
Posted over 1 year ago & has 23 comments

Breakfast Brain Food
Posted over 1 year ago & has 1 comment

Pregnancy Dos and Don'ts
Posted almost 2 years ago & has 8 comments

Body Changes and Discomforts: Itching
Posted over 1 year ago & has 10 comments

Babies Come Into the World Ready To Learn Language
Posted almost 2 years ago & has 1 comment

Practical Ways to Trim High Grocery Prices
Posted almost 2 years ago & has 1 comment

Popular FAQs

How can you tell when you are gaining weight due to the baby growing versus putting weight on your own body?

Why should I buy local produce at farm stands or farmer?s markets? Isn?t the produce at the grocery store from local farmers?

How much water should I drink a day?

What is the recommended breastfeeding goal?

Is air travel safe during pregnancy?

How do different types of caffeine (coffee, tea, soft drinks, energy drinks) affect the human cardiovascular system?

What Are Sleep Deprivation and Deficiency?

Does fruit juice, an 8-ounce glass, count as a fruit serving?

View All FAQs

Resources
The resources section includes local and national listings of services, websites, and information to provide logistical or informational support to pregnant women.

Resources:

HELP What is this?

Categories

- Pregnancy Related Community Organizations
- Smoking Cessation
- Community Organizations For Parents, Infants, Toddlers and Children
- Breastfeeding Resources
- Single-Parent Resources
- Holistic Services and Groups
- Diaper Delivery Resources/Services
- Prenatal Care Providers
- Local Health Clubs and Gyms
- Child Care Referral
- Child Care Centers
- Local Farmers Markets
- Infant and Child Safety Resources
- Maternity Clothes

Most Recent Resources

Principles of Parenting: Sending Messages of Love
Posted about 1 year ago & has 0 comments

From NPR- Hours After A Meal, It's The Memory That Matters
Posted about 1 year ago & has 0 comments

From NPR- Calorie Tracking Apps May Help Boost Weight Loss
Posted about 1 year ago & has 0 comments

From NPR- Mushroom Ice Cream, Anyone? Chefs Turning To Veggies For Dessert
Posted about 1 year ago & has 0 comments

Grocery List Apps That Make Shopping, Syncing Lists Simple
Posted about 1 year ago & has 0 comments

6 Online Tools to Help You Quit Smoking for Good
Posted about 1 year ago & has 0 comments

From NPR- Living On Food Stamps: Newark Mayor Cory Booker Takes Up Challenge
Posted about 1 year ago & has 0 comments

Blogs

Participants used blogs to write about their experiences during pregnancy and to solicit advice from other participants. Blogs were approved by a moderator and a sample entry that was viewable to control and intervention arm subjects is shown below.

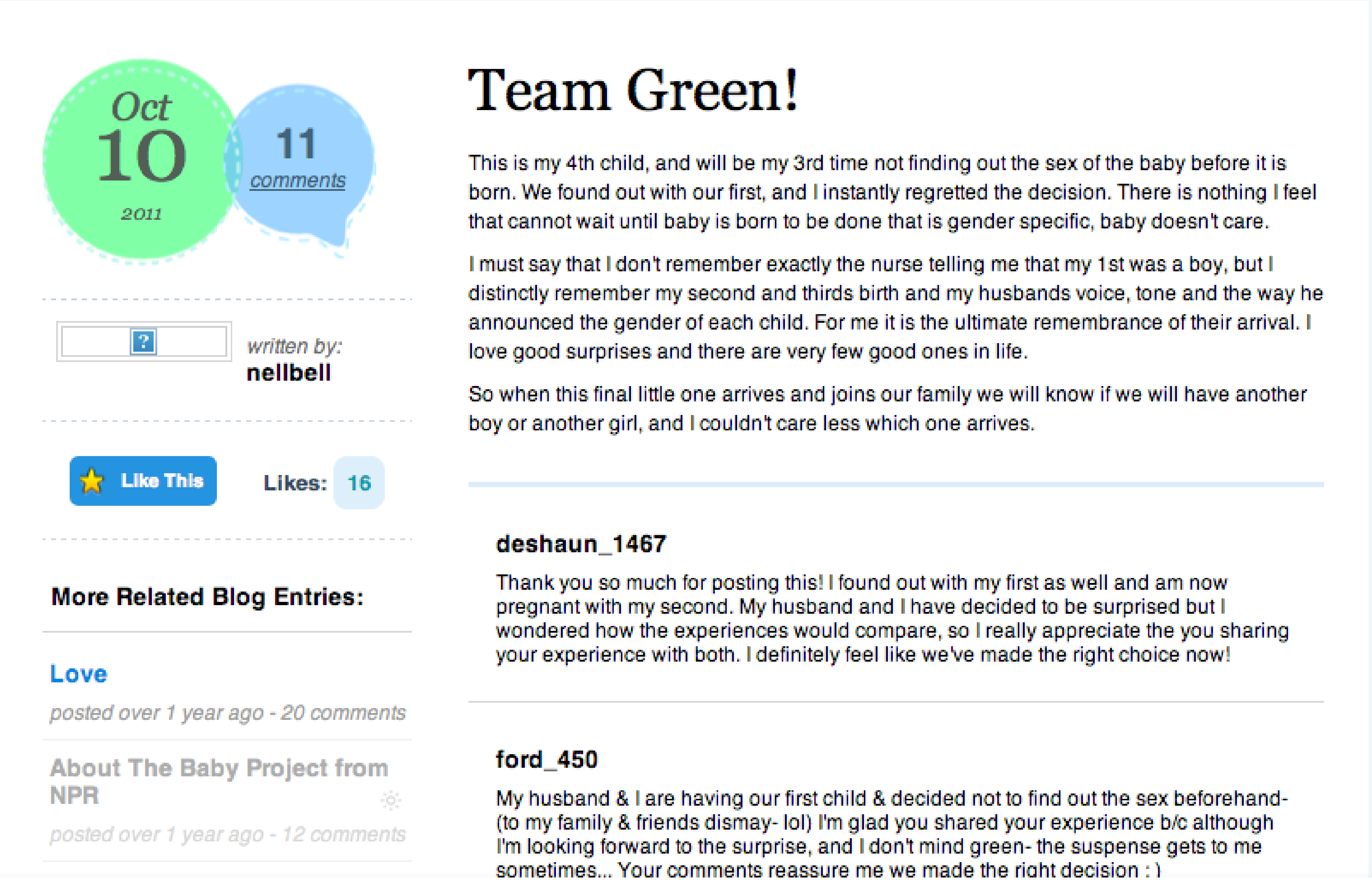

Supplement: Supplementary file 1 [file jmir_v16i8e194_app1.pdf]
